# Near-field effects and energy transfer in hybrid metal-oxide nanostructures

**DOI:** 10.3762/bjnano.4.34

**Published:** 2013-05-14

**Authors:** Ulrich Herr, Barat Achinuq, Cahit Benel, Giorgos Papageorgiou, Manuel Goncalves, Johannes Boneberg, Paul Leiderer, Paul Ziemann, Peter Marek, Horst Hahn

**Affiliations:** 1 Institute for Micro- and Nanomaterials, Ulm University, Albert-Einstein-Allee 47, 89081 Ulm,Germany; 2 Institut für Experimentelle Physik, Ulm University, Albert-Einstein-Allee 11, 89069 Ulm, Germany; 3 Fachbereich Physik, Universität Konstanz, Universitätsstraße 10. 78457 Konstanz, Germany; 4 Institut für Festkörperphysik,Universität Ulm, Albert-Einstein-Allee 11, 89069 Ulm, Germany; 5 Institut für Nanotechnologie, Karlsruhe Institute of Technology, Hermann-von-Helmholtz-Platz 1,76344 Eggenstein-Leopoldshafen, Germany

**Keywords:** confocal microscopy, energy transfer, field enhancement, light harvesting, luminescence, nano-antennas, nanosphere lithography, nanostructures, plasmonics, simulation, TiO_2_ nanoparticles

## Abstract

One of the big challenges of the 21st century is the utilization of nanotechnology for energy technology. Nanoscale structures may provide novel functionality, which has been demonstrated most convincingly by successful applications such as dye-sensitized solar cells introduced by M. Grätzel. Applications in energy technology are based on the transfer and conversion of energy. Following the example of photosynthesis, this requires a combination of light harvesting, transfer of energy to a reaction center, and conversion to other forms of energy by charge separation and transfer. This may be achieved by utilizing hybrid nanostructures, which combine metallic and nonmetallic components. Metallic nanostructures can interact strongly with light. Plasmonic excitations of such structures can cause local enhancement of the electrical field, which has been utilized in spectroscopy for many years. On the other hand, the excited states in metallic structures decay over very short lifetimes. Longer lifetimes of excited states occur in nonmetallic nanostructures, which makes them attractive for further energy transfer before recombination or relaxation sets in. Therefore, the combination of metallic nanostructures with nonmetallic materials is of great interest. We report investigations of hybrid nanostructured model systems that consist of a combination of metallic nanoantennas (fabricated by nanosphere lithography, NSL) and oxide nanoparticles. The oxide particles were doped with rare-earth (RE) ions, which show a large shift between absorption and emission wavelengths, allowing us to investigate the energy-transfer processes in detail. The main focus is on TiO_2_ nanoparticles doped with Eu^3+^, since the material is interesting for applications such as the generation of hydrogen by photocatalytic splitting of water molecules. We use high-resolution techniques such as confocal fluorescence microscopy for the investigation of energy-transfer processes. The experiments are supported by simulations of the electromagnetic field enhancement in the vicinity of well-defined nanoantennas. The results show that the presence of the nanoparticle layer can modify the field enhancement significantly. In addition, we find that the fluorescent intensities observed in the experiments are affected by agglomeration of the nanoparticles. In order to further elucidate the possible influence of agglomeration and quenching effects in the vicinity of the nanoantennas, we have used a commercial organic pigment containing Eu, which exhibits an extremely narrow particle size distribution and no significant agglomeration. We demonstrate that quenching of the Eu fluorescence can be suppressed by covering the nanoantennas with a 10 nm thick SiO*_x_* layer.

## Introduction

Mankind has faced a growing demand for energy at all times in its history, but the 21st century is characterized by the approaching limits to the exploitation of nonrenewable resources (see, e.g., the present “peak oil” discussion). It is generally agreed now that the solution to the problem of supplying sufficient energy to present and future generations lies in techniques for using renewable energies, such as wind, water and direct sunlight. Direct conversion of sunlight by photovoltaics is an extremely attractive method of energy conversion, since the “final product” is supplied already in a most useful form. On the other hand, the storage of energy is a particularly important issue in this case, since the energy supplied by photovoltaics depends on the local and temporal availability of sunlight. There is again general agreement on the fact that the use of renewable energy sources requires reliable and large-scale energy storage. A most attractive way to this end would be conversion of solar energy directly into chemical energy; this can, for example, be achieved by photocatalytic splitting of water into hydrogen and oxygen, as already demonstrated forty years ago [1]. Nanotechnology holds great promises for the development of new devices in the field of advanced energy conversion. This became very apparent with the development of the dye-sensitized solar cells by M. Grätzel [2] more than 20 years ago. Other than conventional semiconductor photovoltaic cells, which depend on a p-n junction for separating electrons and holes generated by photon absorption, these cells are based on the very different mobility of electrons and holes. The electrons are injected into the conduction band of nanostructured TiO_2_, where the nanostructure provides a sufficient contact area between the organic dye molecules and the semiconductor to make the process efficient. The Grätzel cell thus mimics the natural process of photosynthesis, where light harvesting, energy transfer to a reaction center, and conversion to chemical energy by an electron-transfer reaction, take place at different locations in the functional complexes involved. This early success has inspired the scientific community to further elaborate on this type of concept. In particular, it appears attractive to combine structures that interact strongly with light with structures that can transport and store excited states over some time. The strength of the interaction with light is largely dependent on the availability of mobile electrons that may be excited by the electrical field. On the other hand, in systems with a high density of mobile electrons, the excited states may not live long enough to allow conversion or extraction of energy. Plasmonic excitations in metallic nanostructures are a famous example of this. It has been known for decades that the interaction of the electrons of a metal with light can lead to local enhancement of the electrical field, which is utilized in spectroscopy to achieve local sensitivity on the subwavelength scale in Raman spectroscopy and related techniques [3–4]. More recently, the interest of researchers has turned towards applications of plasmonic structures in photovoltaics (for recent reviews, see [5–6]). Possible applications depend on the scattering of light for increasing the absorption, especially in thin-film structures, as well as the exploitation of field enhancements in the near-field region. Another approach reviewed in [6] for “light trapping” in photovoltaics is the excitation of surface plasmon polaritons at the interface between metals and semiconductors. Plasmonic-metal nanostructures are also promising for increasing the conversion efficiency of solar energy directly into chemical energy (see review in [7]), such as in plasmon-enhanced water splitting. These systems depend on the close interaction between metallic nanoparticles and semiconductors. Typically, noble metals are used for the metal nanostructures, since they offer both long term stability and strong resonant enhancements in the range of visible light. A general review of materials aspects in nanotechnology-based approaches in energy technology can be found in [8].

From the examples given, it is clear that the realization of nanotechnology-based approaches to efficient solar-energy conversion depends on a thorough understanding of the individual steps of the energy-transfer processes involved. As already mentioned, these include the absorption process itself, which may be modified by the presence of plasmonic metal structures, but also the transfer of charges (typically in the form of electrons and holes), which may support either an external flow of charge in the electrical circuit connected to the photovoltaic cell, or a chemical reaction in which the energy carrier is formed (e.g., H_2_). Although there are an increasing number of experiments aiming at new advanced energy-conversion systems (see, e.g., [9–10]), further progress depends on a quantitative description and modeling of the individual steps [11]. To this end, we have carried out studies of model systems with well-defined structure and composition of both the metallic and the semiconducting part. In the following, we present results of studies on hybrid nanostructures using regular arrays of nanoantennas formed by lithographic techniques. The optical properties of the metal nanostructures have been characterized by optical techniques, and also modeled by appropriate computer simulations. In a second step, these structures have been combined with oxide nanoparticles that are doped by rare-earth ions, such as Eu^3+^. Materials of this kind are used for light conversion, e.g., in fluorescent lighting and white LEDs (light-emitting diodes). They are characterized by efficient conversion of short-wavelength photons into longer wavelength emission; the large Stokes shift allows one to clearly distinguish between excitation and emission. In the form of nanoparticles, these materials (also termed “nano-phosphors”) allow probing locally the electromagnetic field in the vicinity of the plasmonic metal nanostructures. Also, since nonradiative recombination is an alternative to the radiative emission process in the nanophosphors, we may expect to learn more about the transfer of charge between metal and nonmetal from a possible quenching of the emission of the nanophosphor in the presence of the metal. In this way, the nanophosphor acts as a local probe on the nanoscale for the energy-transfer processes of interest.

The paper is organized as follows. First we describe the preparation of the TiO_2_:Eu nanoparticles used in the energy-transfer experiments, together with their relevant optical properties. Then we report details of the preparation of the Ag nanoantennas. Results of numerical simulations of the electromagnetic field in the vicinity of the nanoantennas are then presented and discussed with respect to the factors influencing the experimental fluorescence measurements. Results of TiO_2_:Eu layers on top of Ag nanoantennas prepared in different ways are presented. To further study the effect of agglomeration and fluorescence quenching, we finally present results obtained using a nonagglomerated commercial organic pigment containing Eu.

## Results and Discussion

### A. TiO_2_:Eu nanophosphors

**Figure 1:**
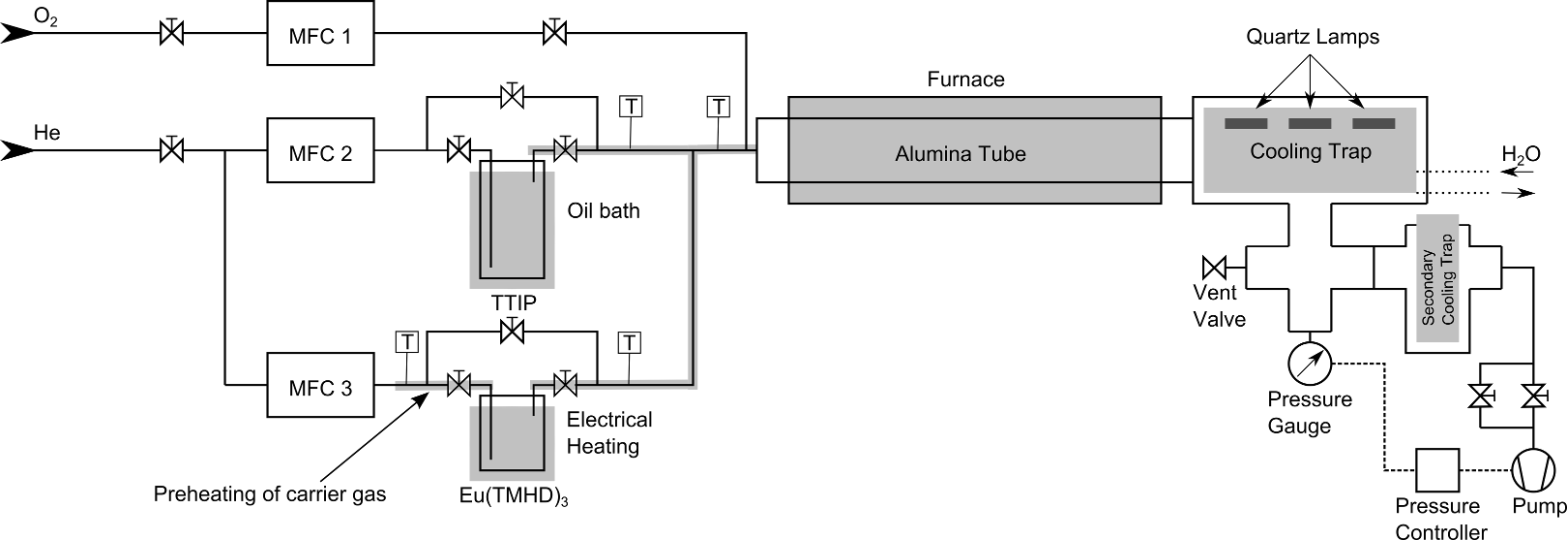
CVR chamber consisting of precursor sources, reaction zone and thermophoretic powder-collection system. The substrates on which the nanoparticles are collected have been placed on the cooling trap below the Quartz lamps.

Nanophosphors can be generated by doping a large-band-gap semiconducting oxide with rare-earth (RE) ions such as Ce^3+^, Eu^3+^ or others. The chemical vapor reaction (CVR) technique has been successfully used for the production of high-purity nanophosphors of this type in the past. Examples include Y_2_O_3_:Eu nanophosphors [12–13] and Y_3_Al_5_O_12_:Ce [14]. In this method, metal–organic precursors are evaporated and reacted with oxygen to form nanoparticles of the desired oxide phase. The particle growth can be controlled by process parameters such as the flow rates of gases, precursor temperature, and furnace temperature (Figure 1). The nanoparticles are carried away from the reaction zone by the gas flow and subsequently deposited inside a powder-collection system. For the generation of hybrid nanosystems, metal nanostructures deposited on substrates such as glass, Si wafers or MgO can be coated by introducing them into the powder-collection system.

For the production of TiO_2_:Eu nanoparticles (NPs), we used titanium(IV) iso-propoxide (TTIP) and tris(2,2,6,6-tetramethyl-3,5-heptanedionato)europiate(III) [Eu(THMD)_3_] (both precursors provided by STREM Chemicals Inc.) at temperatures varying between 45 and 50 °C for the TTIP, and 140 to 160 °C for the Eu(THMD)_3_. The gas flow rates used were 120 mL/min of He and 800 mL/min of O_2_ and the pressure was maintained at 50 mbar by a regulation valve. The furnace temperature was kept at 1000 °C during the process. The composition of the NPs was controlled by energy-dispersive X-ray spectroscopy.

**Figure 2:**
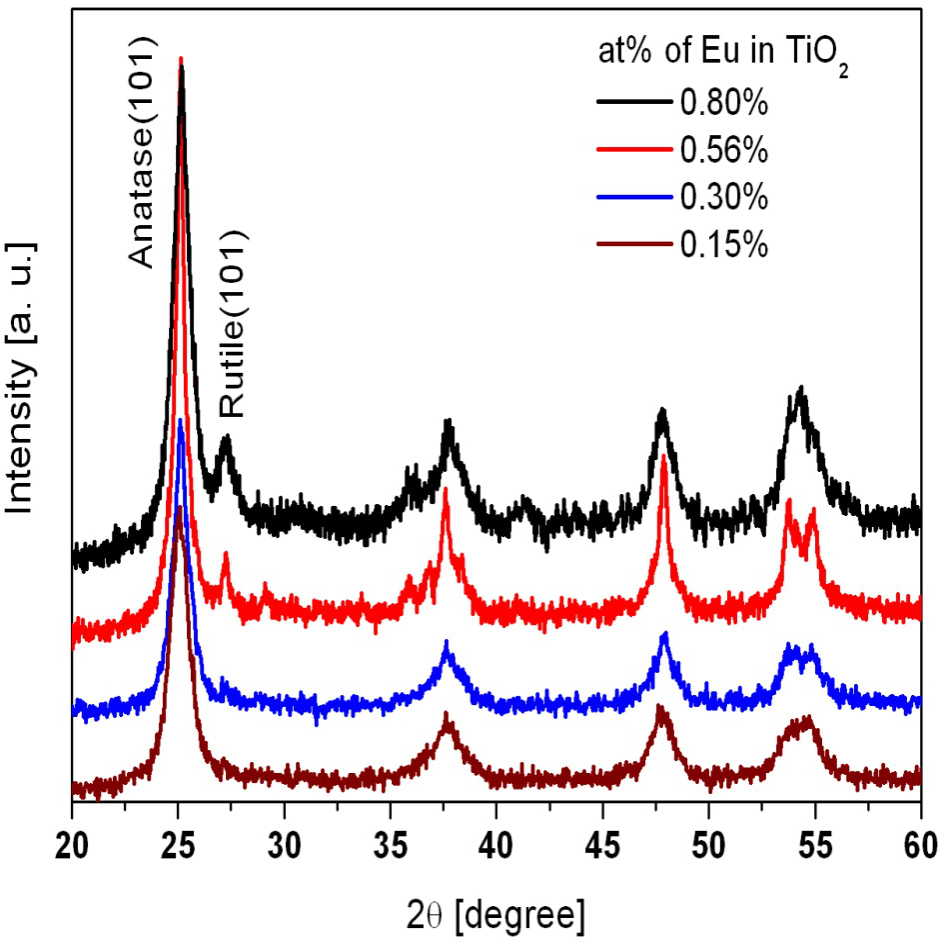
X-ray diffraction patterns (Cu Kα radiation) from the TiO_2_:Eu nanophosphors produced by the CVR method. For low Eu concentrations, only the anatase phase is found, whereas for higher concentrations a mixture of anatase and rutile can be observed.

Figure 2 shows the X-ray diffraction patterns of the as-prepared TiO_2_:Eu NPs with varying composition. For Eu concentrations below 0.56 atom % we find predominantly the anatase structure, whereas for higher Eu concentrations an increasing fraction of rutile is visible.

**Figure 3:**
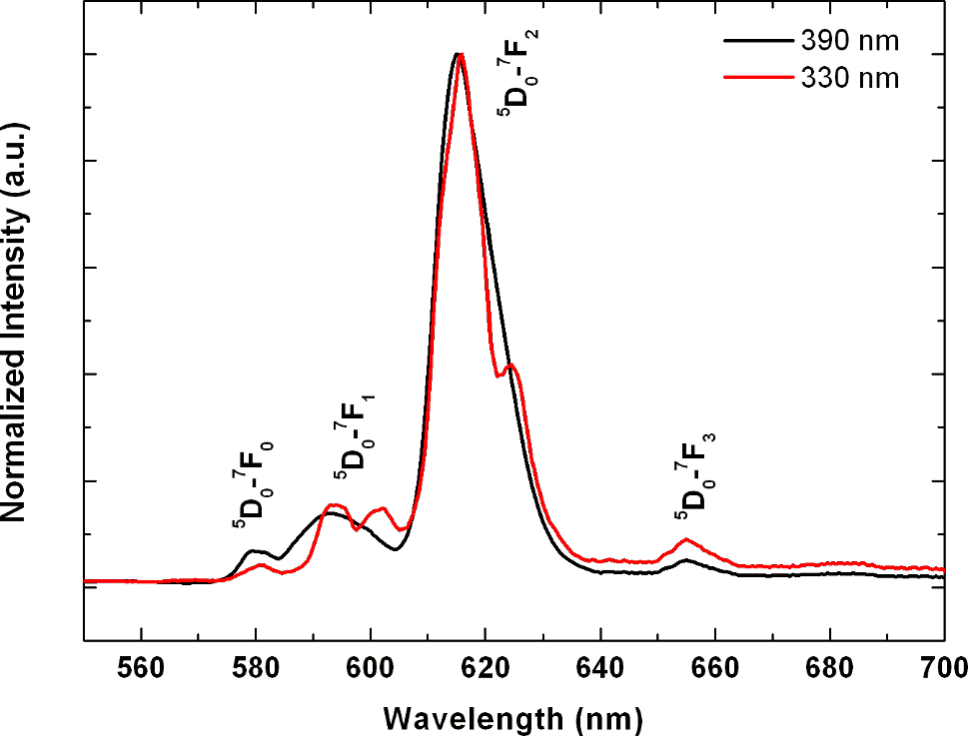
Normalized emission spectra of Eu^3+^ in TiO_2_ under excitation at 330 or 390 nm.

Optical properties of the materials were determined by photoluminescence spectroscopy in a Fluorolog FL3-22 spectrometer (Jobin Yvon) equipped with a Hamamatsu R928P photomultiplier tube. Excitation and emission spectra were measured. Figure 3 shows a typical emission spectrum of a TiO_2_:Eu sample containing 0.8 wt % Eu under excitation at 330 or 390 nm. The characteristic Eu^3+^ emission lines with the dominating ^5^D_0_→^7^F_2_ transition at 617 nm can be clearly observed. For excitation with 330 nm, the photon energy (3.76 eV) is larger than the band gap of the TiO_2_ (3.2 eV), such that excitation through the TiO_2_ host is possible. This may be the reason for the slight differences in the emission at 330 or 390 nm excitation. The emission properties of our TiO_2_:Eu are very similar to the results of Li et al. [15] and Ikeda et al. [16] on Eu-doped TiO_2_ nanoparticles with comparable Eu^3+^ concentration obtained by a plasma-pyrolysis synthesis route.

**Figure 4:**
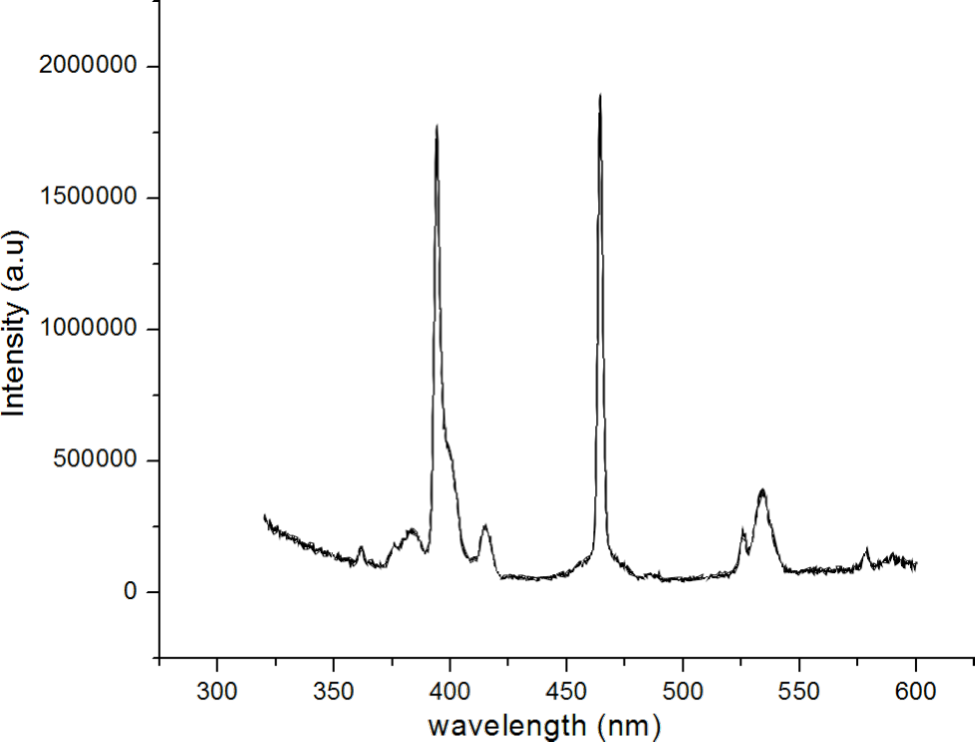
Excitation spectrum of TiO_2_:Eu nanoparticles detected at 617 nm emission.

The excitation spectrum of the TiO_2_:Eu nanoparticles is shown in Figure 4. The emission intensity at 617 nm was recorded and shows several maxima for excitation at around 400 nm as well as maxima at 467 and 538 nm. According to [15], the maxima at 416, 467 and 538 nm can be attributed to the ^5^D→^7^F transitions of the Eu^3+^ ion. The increase of the intensity at short wavelengths (below 390 nm) can be attributed to indirect excitation through the TiO_2_ host lattice [15].

**Figure 5:**
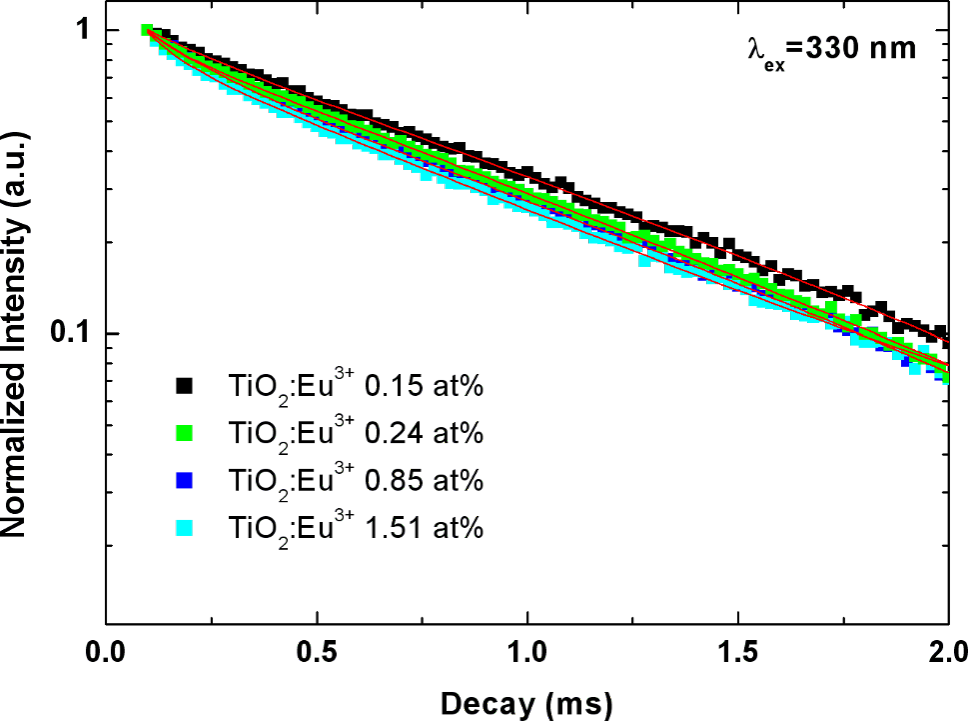
Decay of the TiO_2_:Eu emission intensity with time for excitation at 330 nm.

To learn more about the excitation processes in the TiO_2_:Eu nanoparticles, we conducted time-resolved emission measurements using a xenon flash lamp for the excitation. Figure 5 shows the decay of the emission at 617 nm under excitation at 330 nm. We find that the decay of the emission under these conditions is predominantly exponential with a lifetime of 0.87 ± 0.05 ms, which does not vary significantly with the Eu concentration.

**Figure 6:**
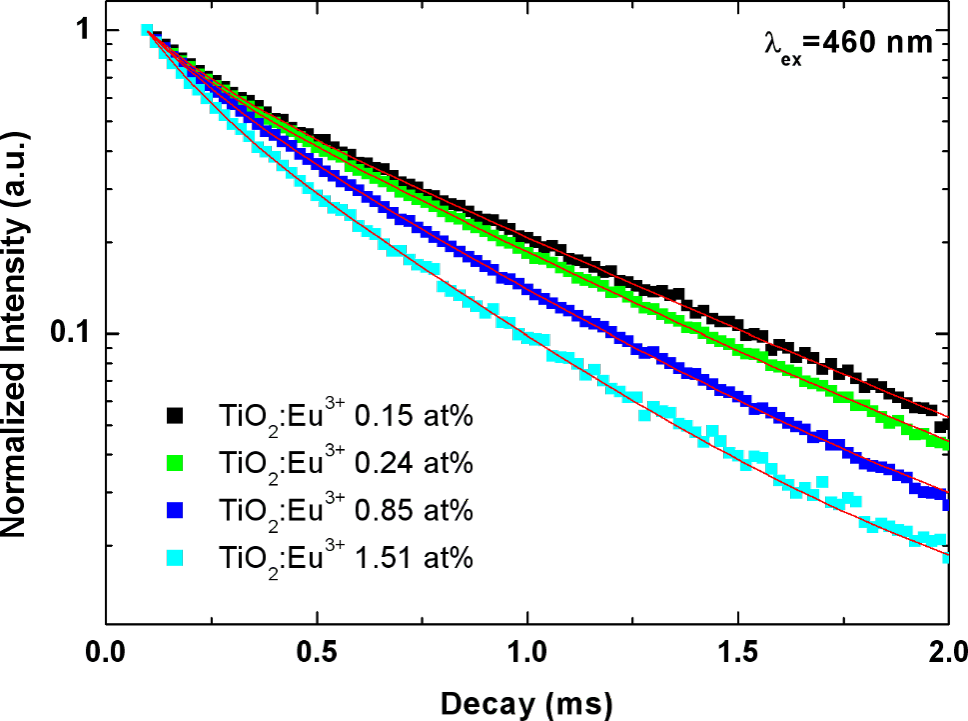
Decay of the TiO_2_:Eu emission intensity at 617 nm with time for excitation at 460 nm.

Different observations are made for excitation below the band gap of the TiO_2_. Figure 6 shows the decay curves measured for excitation with 460 nm wavelength. The curves clearly deviate from a single exponential decay, and the decay behavior clearly depends on the Eu^3+^ concentration. The general trend observed is that the decay gets faster with increasing Eu^3+^ content. The lifetime decreases continuously from 0.69 ms for 0.15 atom % Eu to 0.46 ms for 1.51 atom % Eu. This is a fingerprint of the increasing role of energy transfer between the individual Eu^3+^ ions. Since the excited state of the Eu^3+^ can be depopulated both by radiative processes, but also by nonradiative processes fed by the energy transfer from ion to ion, it is plausible that this transfer leads to a faster depopulation of the excited state, which shows up as a reduction of the lifetime in the present experiments. Similar results have been found in earlier experiments on Y_2_O_3_:Eu produced by the CVR method [13].

**Figure 7:**
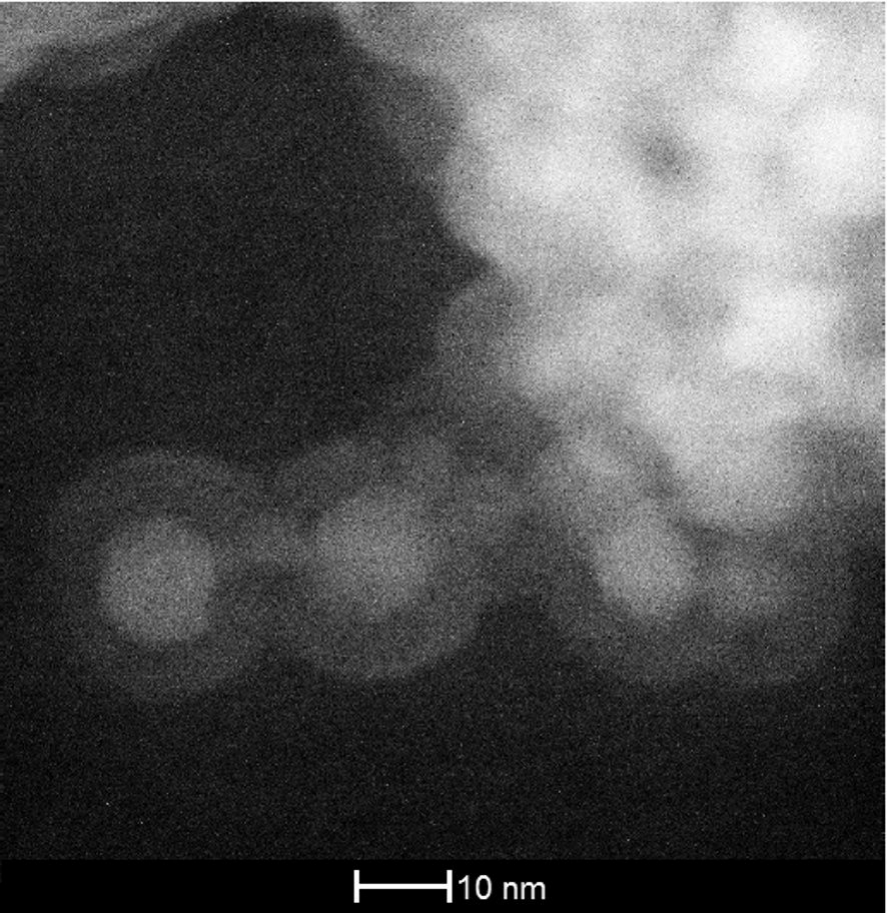
STEM image of TiO_2_:Eu nanoparticles coated with a shell of 3 nm Al_2_O_3_ and TiO_2_.

**Figure 8:**
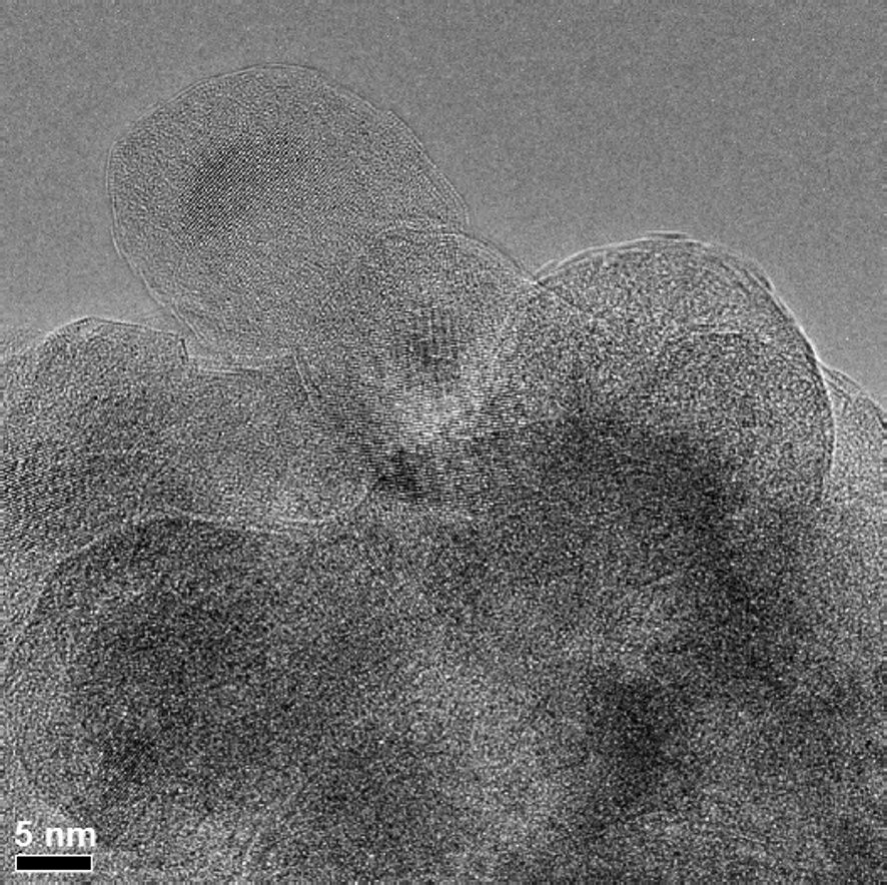
High-resolution TEM image of TiO_2_ nanoparticles coated with Al_2_O_3_ showing that the Al_2_O_3_ coating is amorphous, whereas the TiO_2_ cores exhibit lattice fringes proving that they are crystalline. The length of the scale bar is 5 nm.

These results show that TiO_2_:Eu nanoparticles doped with Eu can be synthesized by the CVR methods, and that they show emission from Eu under different excitation conditions. The ion-to-ion energy transfer (most likely by short-ranged Förster transfer) suggests that the surface may play a significant role, e.g., as a preferred site for nonradiative recombination. The influence of the surface may be investigated by modification of the surface of the nanophosphors. Stable oxides may be considered as a suitable solution for such a coating. In principle, two options may be considered in the present context. A coating with a wide-band-gap oxide material, such as Al_2_O_3_, could passivate the surface and confine excitations inside the TiO_2_ core, similar to techniques applied in core–shell semiconductor nanoparticles. Another option would be a coating with undoped TiO_2_. The lack of Eu in this outer coating shell would effectively suppress the energy transfer to the particle surface, while not disturbing the lattice structure of the TiO_2_:Eu core due to the perfect lattice matching possible in the homoepitaxial case. In order to investigate the possibility for generation of such coatings, TiO_2_:Eu nanoparticles were subjected to a post-processing step in an atomic layer deposition (ALD) chamber supplied with trimethyl aluminium (TMA) and TTIP sources. In both cases, combination with water vapor allows to grow oxides of the respective metal in a layer by layer mode, and achieve a conformal coating of well-defined thickness in this way. The advantage of the ALD process is that this conformal coating can be achieved without using organic surfactants. Results of the ALD post-processing of TiO_2_:Eu nanoparticles are shown in Figure 7 and Figure 8. Figure 7 shows a STEM image of TiO_2_:Eu nanoparticles coated with 3 nm of Al_2_O_3_. In addition, a thin layer of TiO_2_ was added at the end of the process in order to test the possibility of generating multishell structures. One can clearly distinguish the TiO_2_:Eu core from the shell in Figure 7, but it is not possible to distinguish between the Al_2_O_3_ and the TiO_2_ part of the shell.

Figure 8 shows a high-resolution TEM image of TiO_2_ nanoparticles coated with Al_2_O_3_. Lattice fringes can only be observed in the core of the particles, indicating that the TiO_2_ cores are crystalline. However, the absence of lattice fringes in the region of the Al_2_O_3_ shells indicates that the shell is amorphous. The results prove that it is possible to generate potentially passivating coatings by ALD post-processing. However, it has also become obvious that already existing agglomerates will be coated as a whole, therefore not leading to a separate protection of the surface of individual particle. This may be avoided by coating of the particles while they are still being carried in the gas flow of the CVR process. Future work will therefore aim at an integration of the ALD process into the CVR technique.

### B. Systems with Ag nanoantennas from NSL

**Figure 9:**
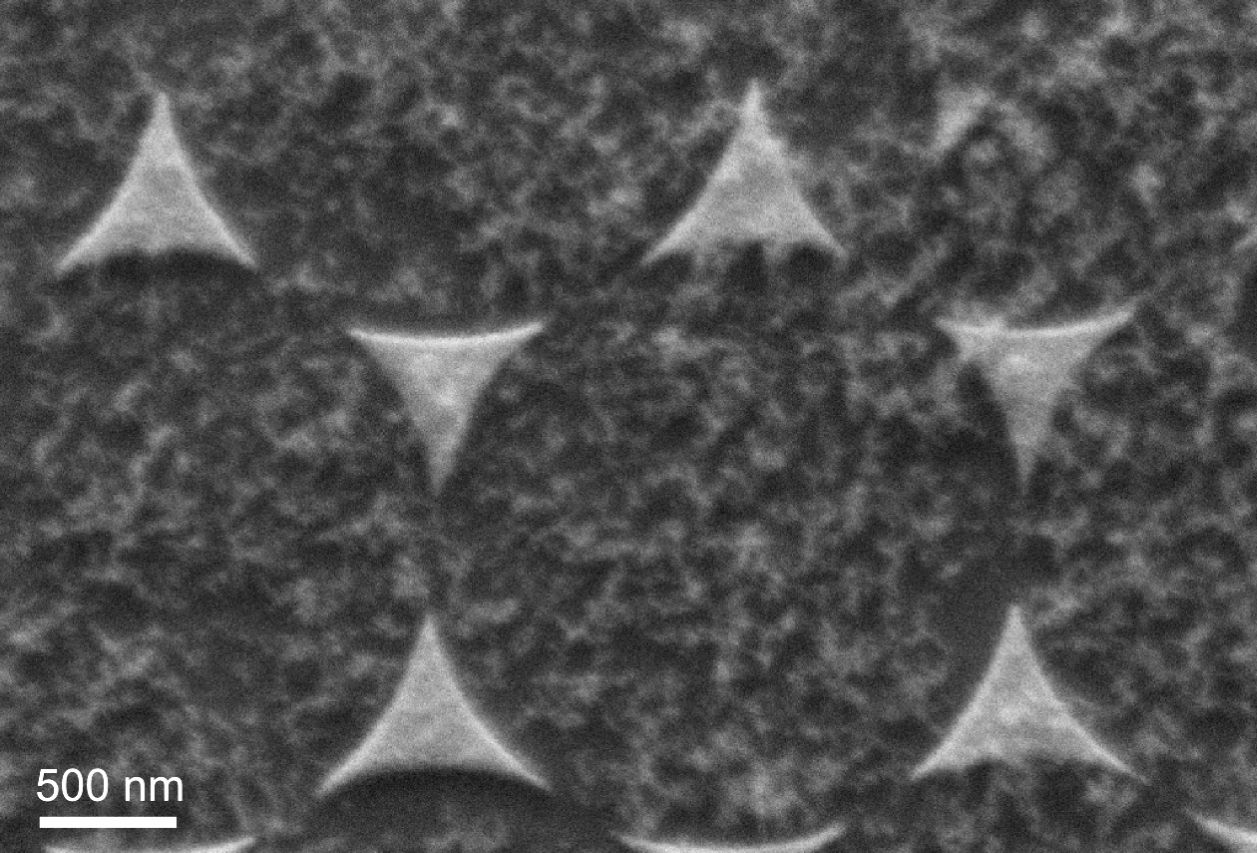
SEM image of Ag nanoantennas from the nanosphere lithography process (using colloid spheres with 3 μm diameter), covered with TiO_2_:Eu nanophosphors.

Nanosphere lithography (NSL) is a well-established technique for the generation of periodic structures on the submicron scale [17]. Colloid spheres, which are commercially available, e.g., as size standards in microscopy, are deposited as part of a suspension onto substrates. After drying, hexagonal close-packed arrangements of the spheres can be obtained, with periodicities extending over quite large distances. Single layers of such spheres serve as template masks for deposition of materials through the open holes of the layer structure. After removal of the colloid particles, typically arrangements of triangular structures are obtained. The distance between the structures is determined by the size of the original colloid sphere, and can be adjusted over a wide range by the choice of the sphere size. In our study, we have used polystyrene spheres with diameters of 1 μm or 3 μm. The water-based dispersions were dried under constant temperature and air flow to assure a constant evaporation rate of the solvent. In the next step, we deposited Ag films with a thickness of 50 nm by thermal evaporation under vacuum conditions. The spheres were removed afterwards by sonication in ethyl methyl ketone and water. The resulting structures are triangular with a regular spacing over wide regions (the typical size of the ordered regions was several 10 μm). These Ag nanoantennas are then covered with nanophosphors in a second processing step. Figure 9 shows a SEM image of a structure obtained by using 3 μm diameter spheres. The cover layer of TiO_2_:Eu nanoparticles was obtained by incorporation of the nanoantenna covered substrate into the powder-collection stage of the CVR machine (see Figure 1).

**Figure 10:**
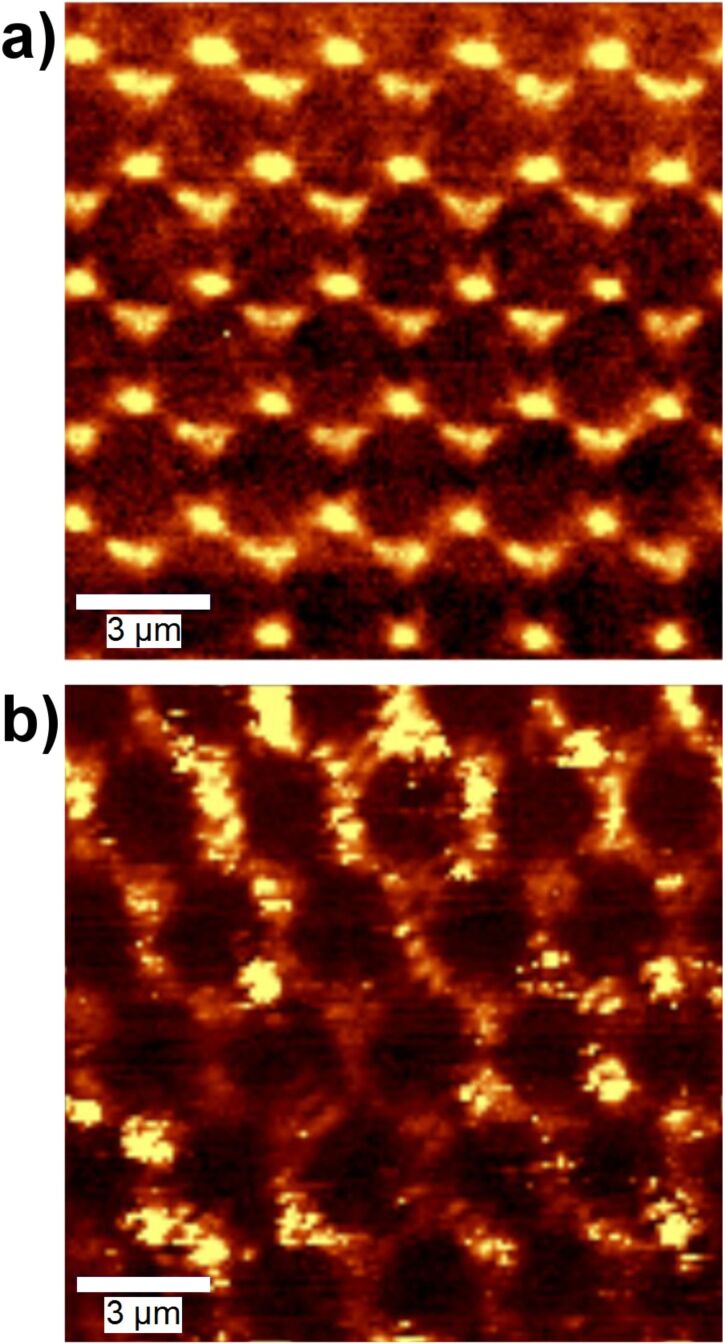
Confocal microscopy images of the Ag nano-antenna structures (produced using 3 μm diameter colloid spheres) covered with TiO_2_:Eu nanophosphors under illumination with 532 nm laser light. In (a) intensity in the wavelength region 530–535 nm has been used, whereas in (b) the region 605–630 nm is used. The scale bars mark a length of 3 μm.

A big advantage of the regular nanoantenna patterns generated in this way is that the positions of the individual antennas can be identified in confocal microscopy, even though the details of the antenna itself may not be resolved. We have used confocal microscopy with excitation by 532 nm laser light to study the optical properties of the nanophosphor-covered Ag nanoantennas. We analyze the light coming from the structure using a spectrometer with a CCD sensor. In this way, we can distinguish between the scattered and reflected primary light, and the fluorescence generated by conversion of the excitation light to photons with 618 nm from the Eu^3+^ ions. Figure 10a and Figure 10b show two confocal microscopy images of the same area of the sample. Figure 10a was obtained by using the wavelength region of 530–535 nm, which contains mainly elastically or quasi-elastically scattered light. In contrast, Figure 10b was obtained using the wavelength region of 605 to 630 nm, where the emission from the Eu^3+^ is concentrated. Several observations can be made from these images. Firstly, the scattered light is concentrated at the nanoantenna structures, with dark areas between the nanoantennas. Secondly, fluorescence occurs all over the sample, but the intensity is higher in the vicinity of the nanoantennas.

The results demonstrate that the regular nanoantenna patterns from NSL offer excellent opportunities for assessment of the coupling of the nanophosphors to the nanoantennas with submicron resolution. However, in order to optimize the resolution it will be necessary to obtain a well-defined arrangement of the phosphor nanoparticles. As can be observed from Figure 9, agglomerates of the nanophosphor do still occur, which will not allow a quantitative measurement of local intensities, since these intensities will not only be determined by the local electrical field strength, but also by the local density of nanophosphor. Therefore, we have investigated alternative ways for deposition of the nanophosphor with the aim of getting more homogeneous particle arrangements. These studies will be reported in section D.

On the other hand, the actual intensity distribution in the near field of the nanoantennas will depend not only on the size and thickness of the antennas, but also on the dielectric properties of its environment, i.e., both the substrate and the nanophosphor cover layer. In addition, the local arrangement of individual nanoantennas with respect to each other (e.g., the gap width between the tips of neighboring antenna triangles) will play a role for the intensity distribution, as well as the orientation towards the plane of polarization of the illuminating light. These effects have been further explored by numerical simulations of the electromagnetic field in model structures, which will be presented in the next section.

### C. Numerical simulation of the electrical field distribution

**Figure 11:**
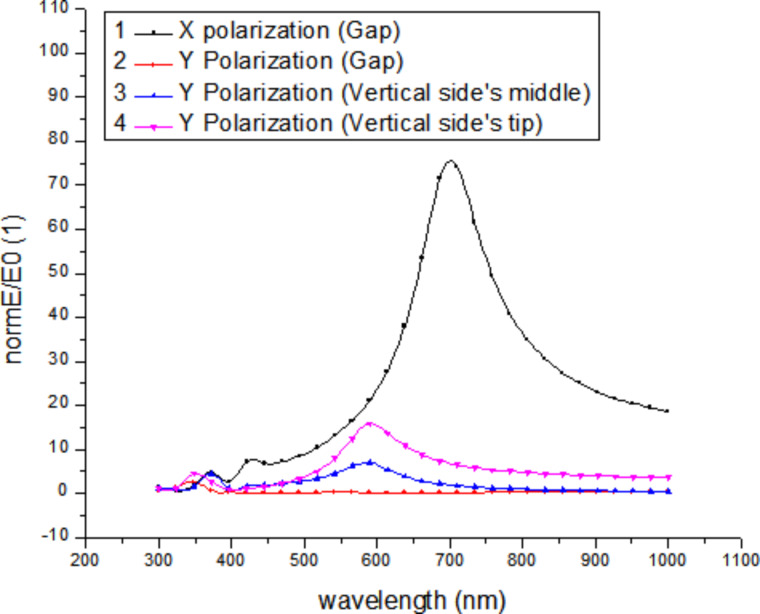
Calculated field-enhancement factor (normalized to the amplitude *E*_0_ of the incident light) for bowtie nanoantennas at different positions and for different polarization of the incident light.

**Figure 12:**
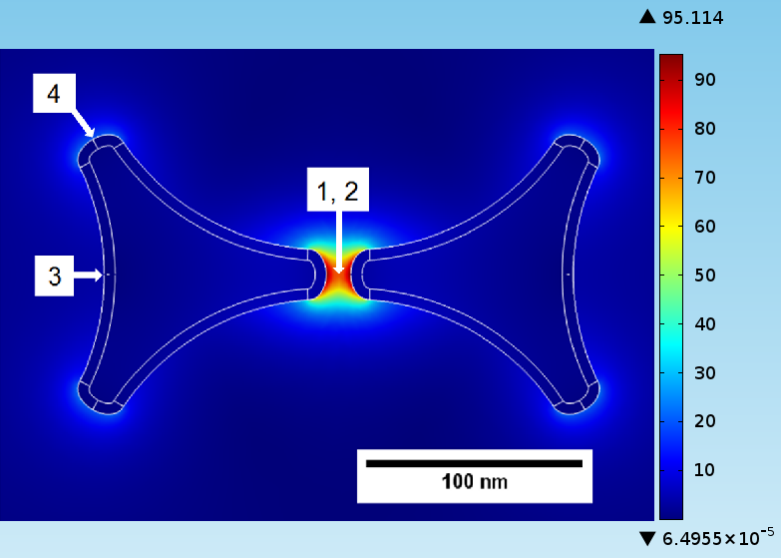
Field enhancement ratio (scale goes from 0 to 90) for Ag bow-tie nano-antennas with tip-to-edge length of 100 nm, thickness 30 nm, and gap size of 12 nm under excitation at 700 nm. The labels 1 to 4 mark the positions for which the field-enhancement curves shown in Figure 11 were calculated.

Numerical simulations have been performed using the COMSOL Multiphysics RF module, which solves the Maxwell equations in the frequency domain based on the finite-element method. Bowtie model antenna structures have been defined with geometries close to the experimental ones. The aim was to calculate the relative electrical field enhancement factor *E*/*E*_0_ of the local field strength *E* over the field of the incident light wave *E*_0_. Dielectric properties of Ag have been taken from the literature [18]. As an example, Figure 11 shows the field-enhancement factor in the center of the gap between two triangles of a bowtie nanoantenna structure made of a 30 nm thick Ag layer for two different polarizations (the long axis of the bowtie is along the *x* direction). For comparison, field enhancement factors for two other positions outside the gap region are shown in the same image. The size of the gap is 12 nm, and the tip-to-edge length of the antenna arms (measured from the tip of the triangle to the centre of the opposing base) is 100 nm. Resonances are found, which depend on the size of the antenna but also on the dielectric constants of the environment (substrate and cover layer). The spatial distribution of the enhancement factor in the region of the maximum (around 700 nm) for polarization in the horizontal (*x*) direction is shown in Figure 12.

**Figure 13:**
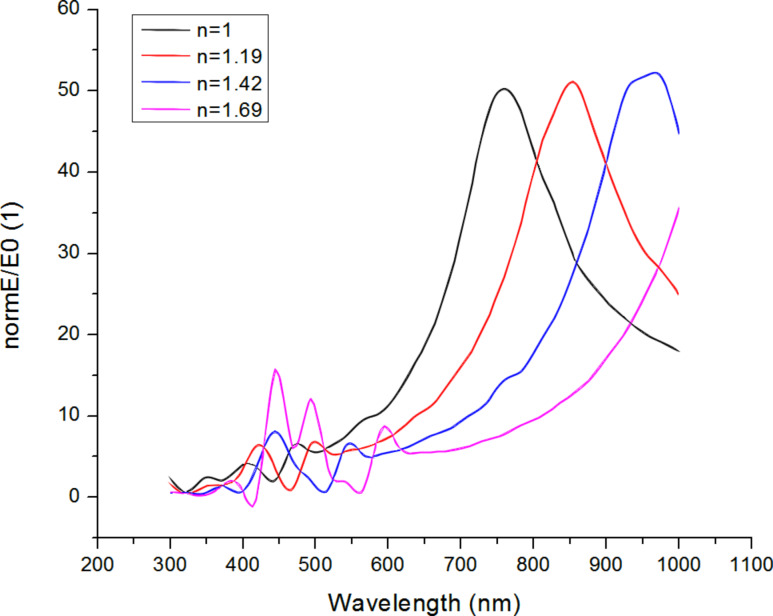
Dependence of the field enhancement in the centre of the gap of a bowtie antenna structure on the index of refraction (*n* = ε^0.5^) of the cover layer. The substrate is glass (*n* = 1.54).

The dielectric constant ε of the environment depends on the density of the TiO_2_ particles in the cover layer. To get an idea about the importance of this effect, we have simulated cover layers with varying average dielectric constant (using an effective-medium approach) and thickness, which aims at mimicking the effect of different packing densities of TiO_2_ powder particles in the cover layer. As an example, Figure 13 shows results for the field-enhancement factors in the gap center for a variation of the index of refraction *n* (we can use *n* = ε^0.5^ here).

The simulation results show that the arrangement of the nanophosphor particles in the cover layer is important, since the presence of the nanoparticles has a significant effect on the resonant behavior of the nanoantennas. For a fixed excitation wavelength, the field enhancement factor can vary locally with the local density of the nanophosphor. It is therefore most important to achieve a regular and reproducible arrangement of the nanophosphor inside the cover layer.

**Figure 14:**
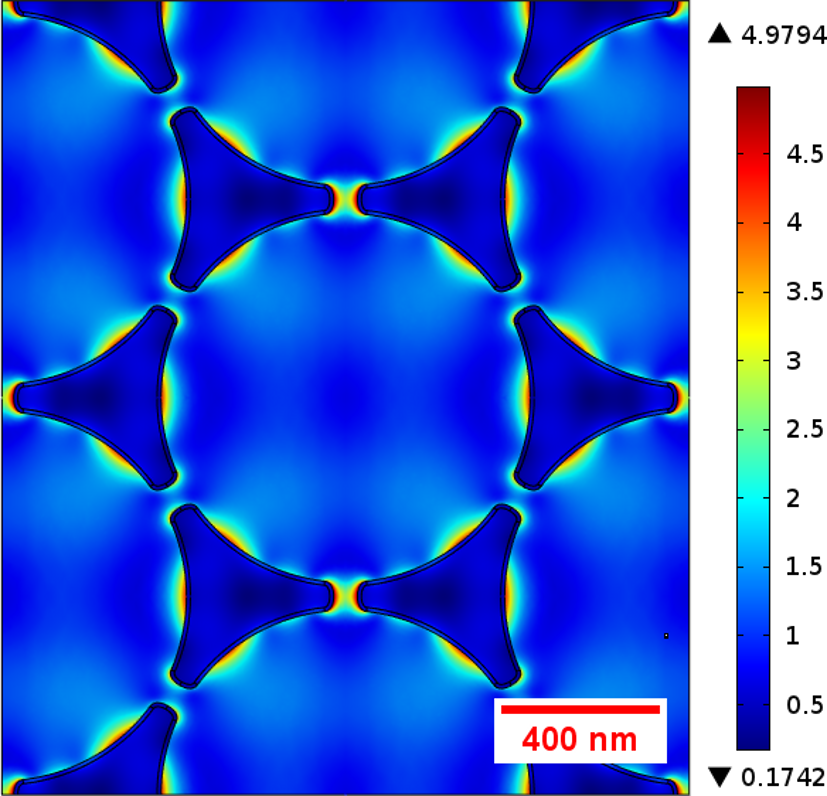
Field enhancement ratio for an array of Ag nanoantenna structures with tip-to-edge length of 370 nm, thickness of 50 nm and gap size of 60 nm calculated for excitation at 532 nm. The polarization direction of the electrical field is horizontal.

Another result of the simulations is that the maximum field enhancement that can be achieved depends on the size of the gap between the antennas. Since the structural dimension and the gap size scale with the diameter of the colloid spheres, the use of smaller spheres is more promising. However, for very small sphere sizes it gets increasingly difficult to identify the positions of individual antennas in the confocal microscope. As a compromise, we chose polystyrene spheres with 1 μm diameter for the following experiments. To obtain realistic values for the expected field enhancements, we performed a simulation of an array of 50 nm thick Ag nanoantennas with tip-to-edge length of 370 nm and a gap size of 60 nm. The individual antenna dimensions correspond to the dimensions of the experimental antennas we obtained when using 1 μm diameter spheres. The results are shown in Figure 14. One can observe significant field enhancement in the gap region, but also at other regions of the individual antennas. Considering the limited resolution of the confocal microscope, we can expect to see the antennas as bright regions in the fluorescence images.

### D. Optimization of the nanophosphor layer

**Figure 15:**
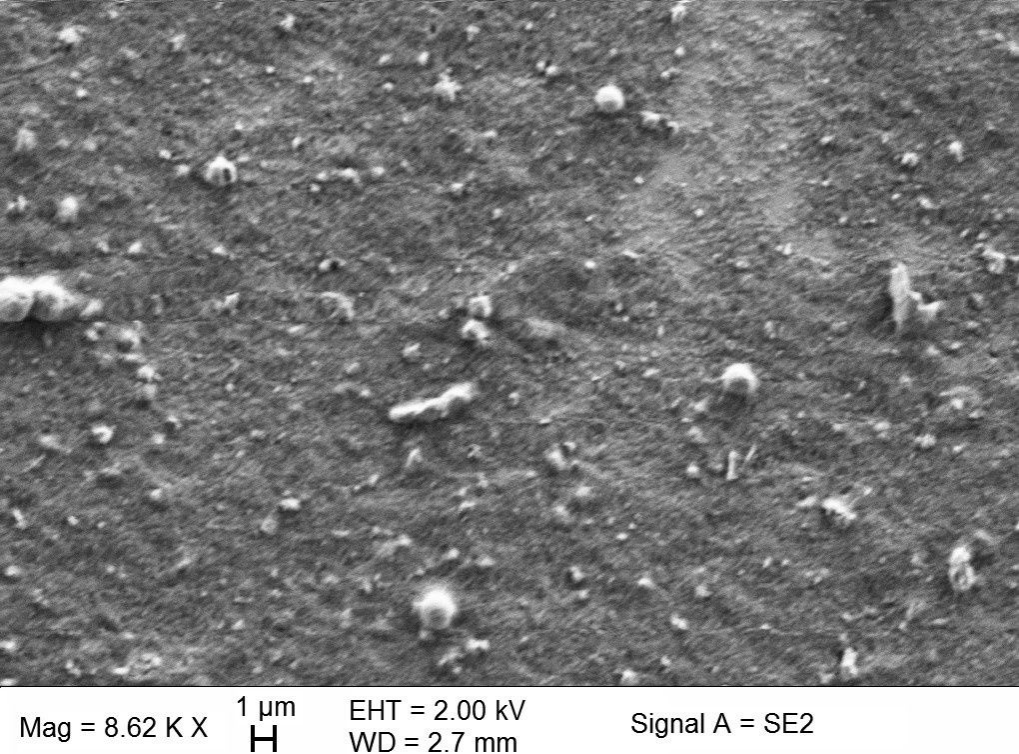
SEM image of spin coated TiO_2_:Eu layer on Ag nanoantenna structures.

**Figure 16:**
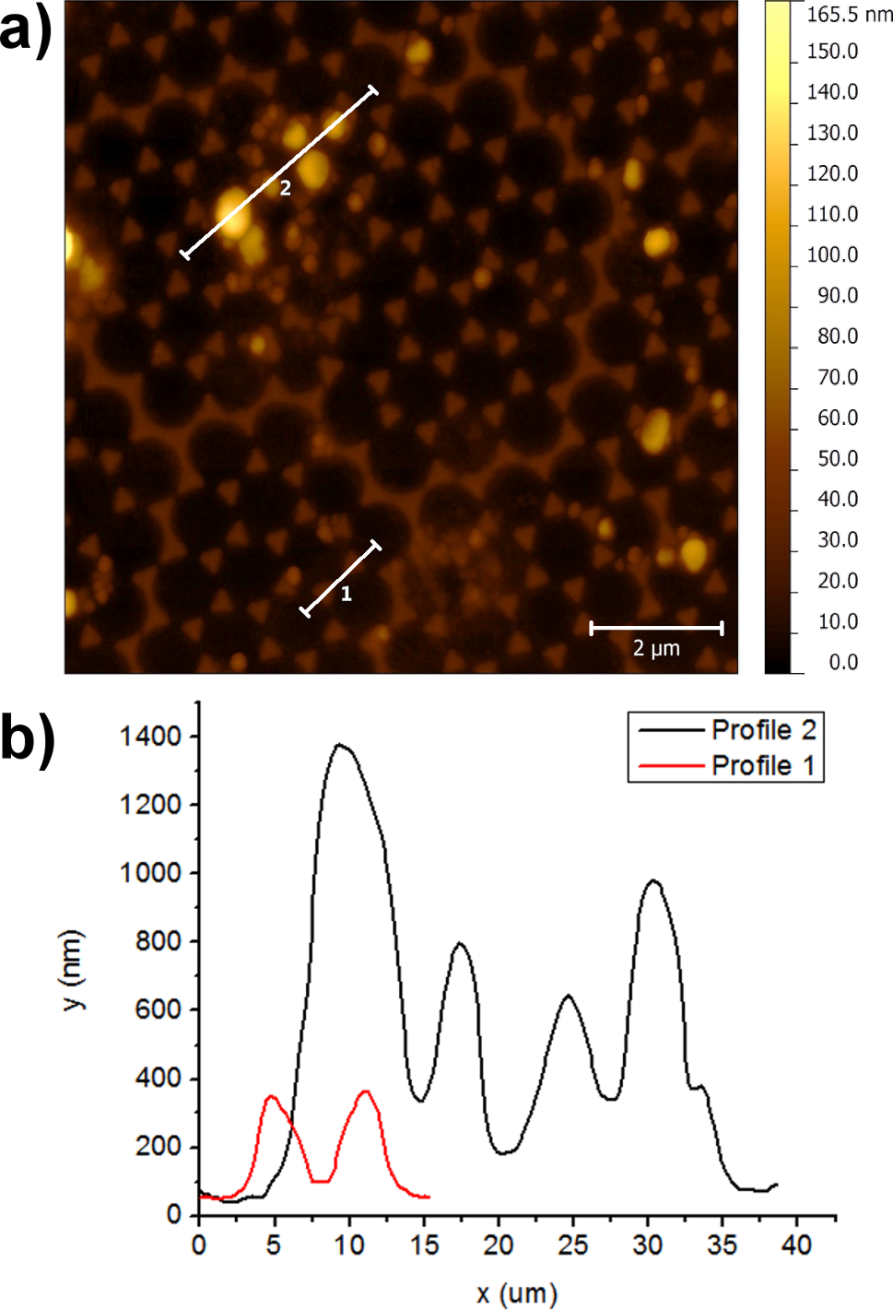
(a) AFM image of the spin coated TiO_2_:Eu layer. The antenna structure is still visible in this region. (b) AFM height profiles along the lines marked in (a) (black line corresponds to line 2 in (a), red line to line 1 in (a)).

In order to homogenize the distribution of the TiO_2_:Eu nanoparticles in the cover layer, the nanoparticles collected from the CVR process were dispersed in ethanol solution (95 % ethanol, 5% water), mixed and sonicated with ultrasound. The nanosuspensions obtained in this way were spin-coated on top of glass substrates covered with Ag nanoantennas produced by NSL at a speed of 2000 rpm and a spinning time of 10 s. Figure 15 shows an SEM image of the spin-coated TiO_2_:Eu particles. The surface is characterized by regions of relatively homogeneous particle deposition, but also by the presence of large particles. Good results by confocal fluorescence imaging can only be expected from the smooth regions. Figure 16 shows an AFM image of a smooth region. From the image in Figure 16a and the line profiles in Figure 16b it can be observed that even in the smooth regions agglomerated particles with sizes of more than 100 nm can be found.

**Figure 17:**
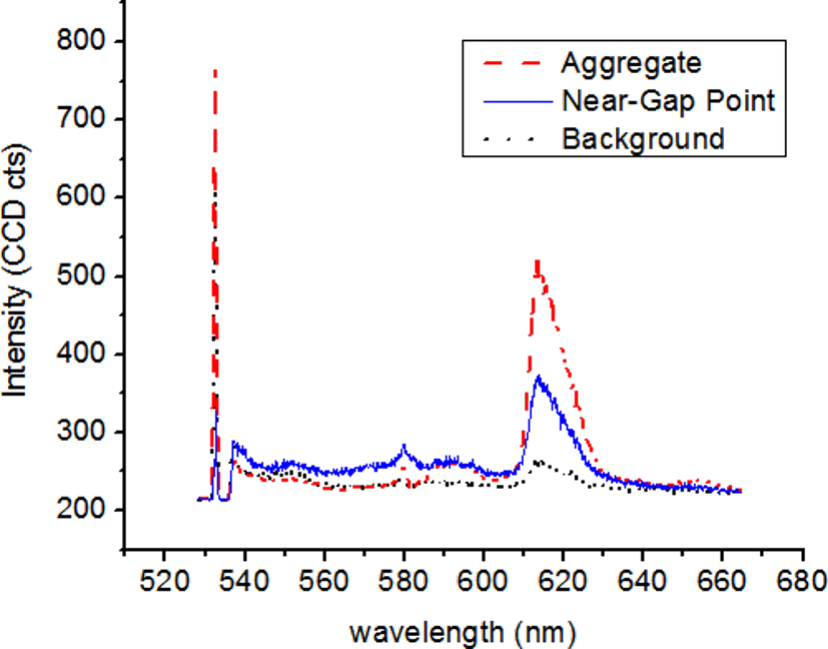
Fluorescent intensity obtained by confocal microscopy of spin-coated nanoantenna structures under excitation with 532 nm radiation.

The effect of agglomerates becomes evident by comparison of fluorescent intensities from different areas of the sample. Figure 17 shows the intensities taken from a region with a large aggregate (red line), a point near the gap of a bowtie nanoantenna (blue line), and a region without nanoantennas (background intensity, black line). It is observed that the intensity of the large aggregate is higher than that from the near-gap point. This result makes the interpretation of fluorescent intensity enhancements quite difficult. We can conclude here that although fluorescence intensity enhancements are observed in the vicinity of the Ag nanoantennas, a quantitative interpretation in terms of local field enhancement will require further optimization of the nanoparticle arrangement.

### E. Study of quenching effects using organic pigments

**Figure 18:**
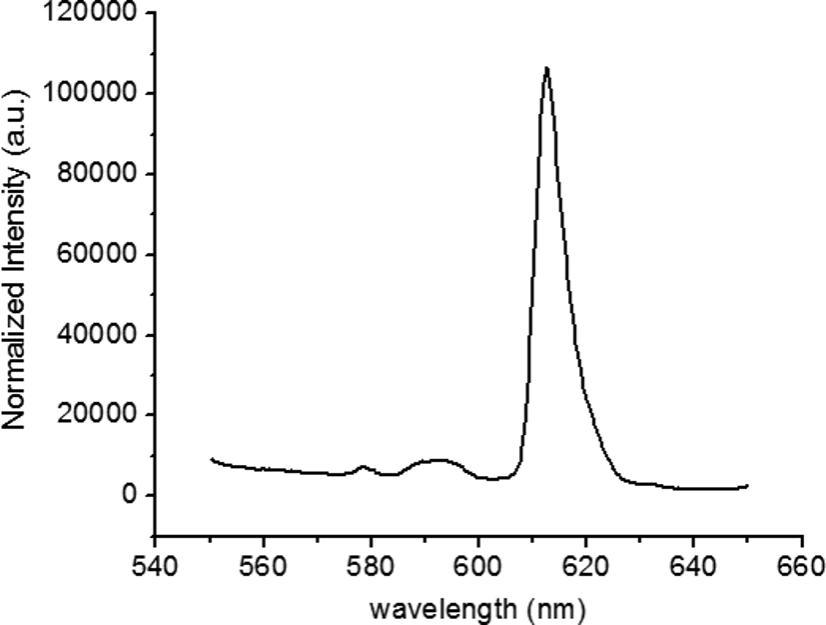
Emission spectrum of VTLUNP organic pigment under excitation with 532 nm radiation.

**Figure 19:**
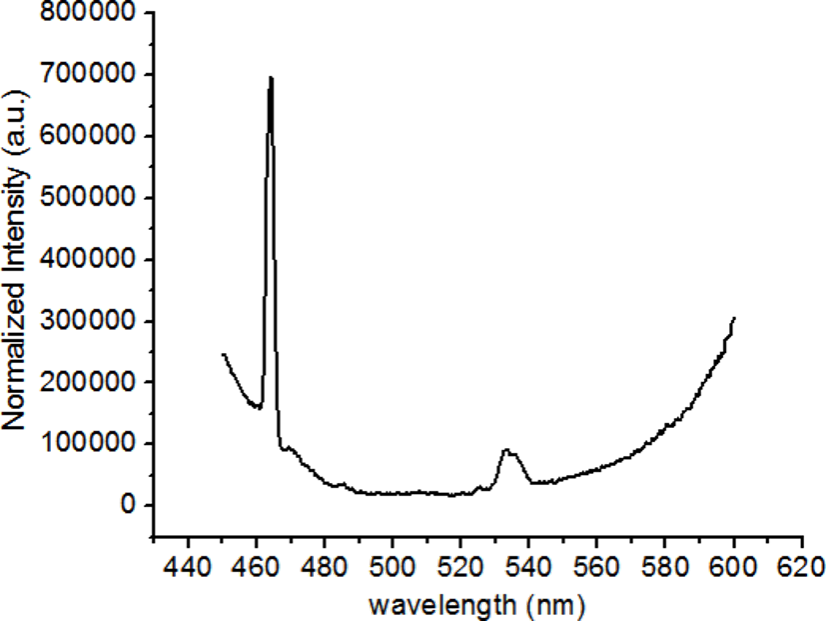
Excitation spectrum of VTLUNP organic pigment for emission at 614 nm.

**Figure 20:**
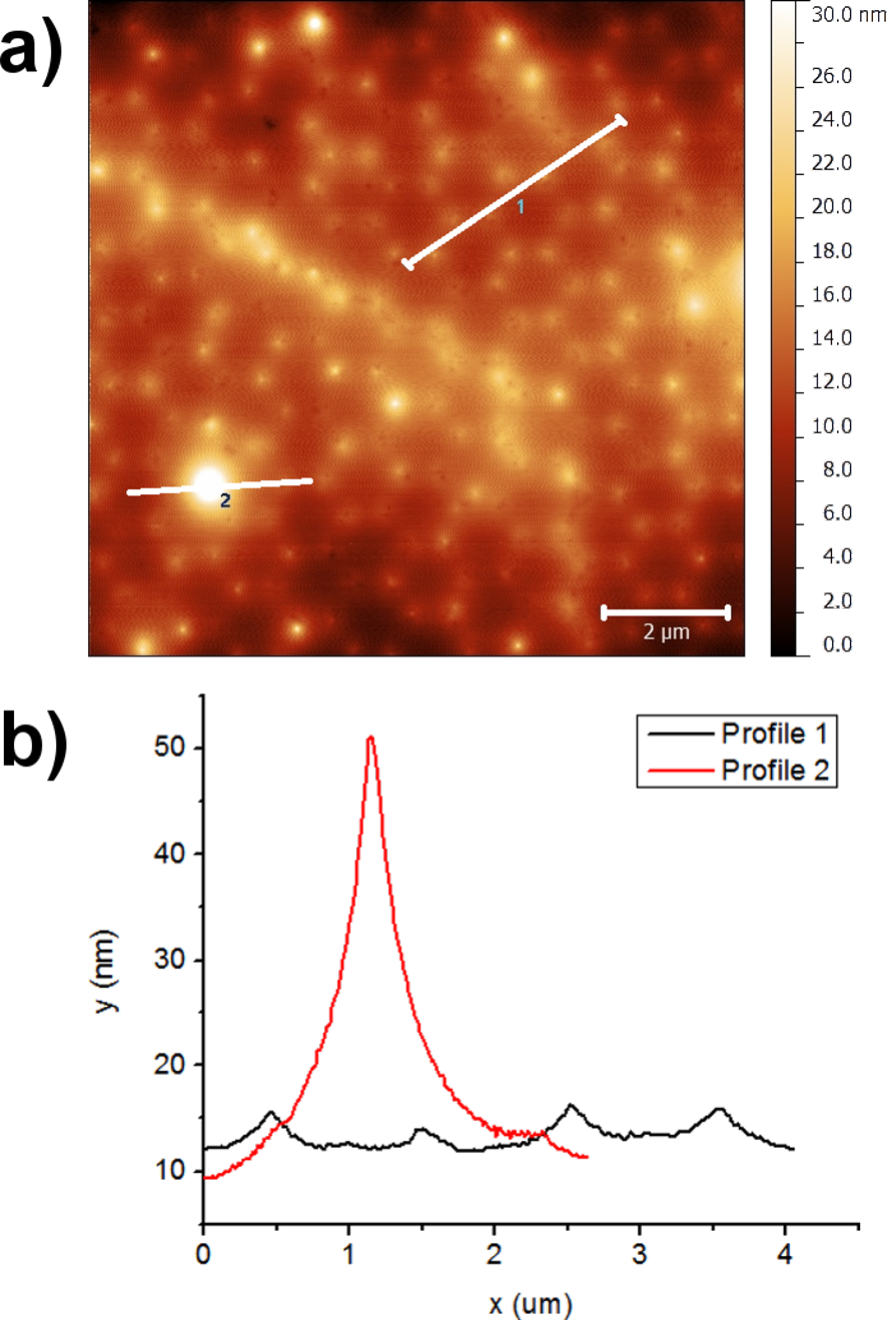
(a) AFM image of Ag nanoantennas spin coated with VTLUNP (b) AFM height profiles along the lines 1 and 2 marked in (a).

The results reported up to know show the importance of obtaining a narrow distribution of nanoparticle sizes. This can be achieved by stabilizing the nanoparticle dispersions with surfactants. In this part of the study, we used a nanosuspension based on a commercial fluorescent organic pigment (VTLUNP by LuminoChem), which exhibits excitation and emission spectra very similar to the ones of the TiO_2_:Eu. The luminescent center in this pigment is Eu. The emission spectrum for excitation with 532 nm radiation is shown in Figure 18. A strong red emission with a maximum at 614 nm is observed. The excitation spectrum recorded with the 614 nm emission is shown in Figure 19. Similar to the TiO_2_:Eu, it shows maxima at 465 nm and 535 nm, which can be attributed to the Eu. The commercial nanosuspension exhibits a narrow particle size distribution centered around 23 nm, as confirmed by dynamic-light-scattering measurements. The nanosuspensions were spin coated at 2000 rpm for 10 s onto Ag nanoantennas. AFM scans of the coated samples show smooth surfaces with roughness values of a few nanometers (see Figure 20). In contrast to the spin-coated TiO_2_:Eu solutions, no large aggregates have been observed. The samples therefore seem appropriate for studying the local field enhancement around the nanoantennas.

**Figure 21:**
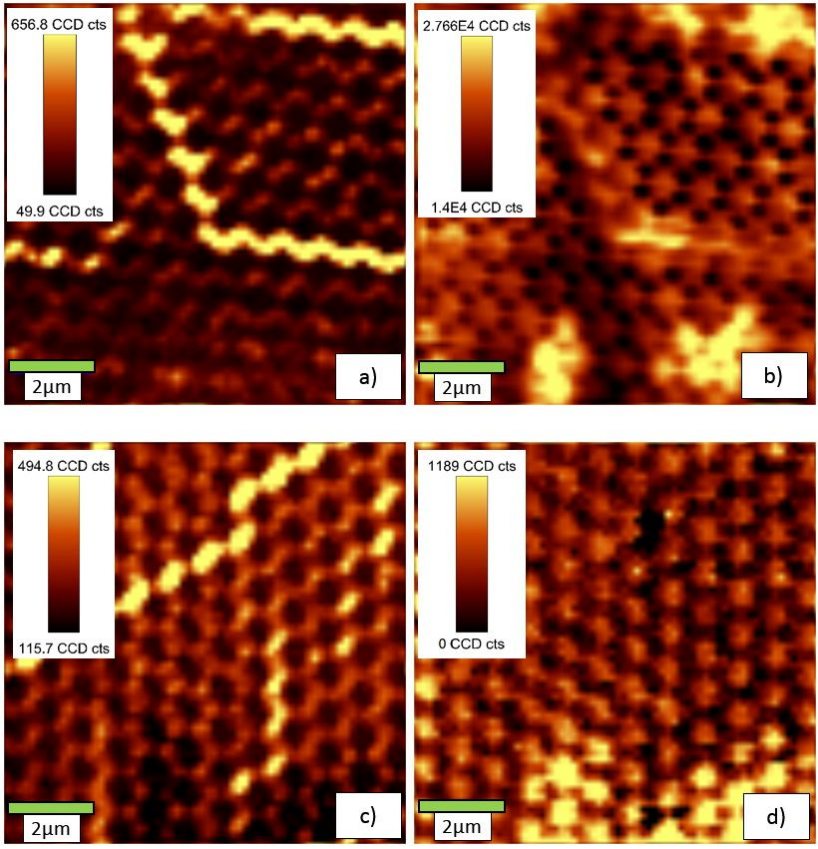
Representative scattering (a, c) and fluorescence (b, d) images of the samples spin coated with VTLUNP nanosuspensions. Areas of high film coverage (a), (b) and low film coverage (c), (d) are depicted.

Figure 21 shows results of samples with high (a, b) or low (c, d) film coverage. For each case, a direct comparison of scattered light in the wavelength region around the excitation wavelength (Figure 21a and Figure 21c) and red fluorescence intensity (Figure 21b and Figure 21d) is made. From this comparison, we find that the areas right above the nanoantennas (bright spots in Figure 21a and Figure 21c) appear dark in the fluorescence images (Figure 21b and Figure 21d). Also, the circular areas enclosed in between the nanoantennas (which were covered by the colloid particles during Ag evaporation) appear bright in fluorescence. Both observations seem to contradict the expectations from the simulations. In principle, the observations may be explained by inhomogeneities in the phosphor layer thickness, which might be associated with local variations of the wetting behavior caused by the Ag nanoantennas. Another possible explanation is a quenching of the excitation at the metallic surface. Such a quenching of the fluorescence has been reported earlier for noble-metal nanospheres [19–20].

The quenching can be suppressed by inserting a dielectric layer between the metal and the semiconducting nanophosphors. This can either be achieved by coating of the nanoparticles, or by deposition of a dielectric layer on the metal nanoantennas. We tried the second solution by coating of the Ag nanoantennas with a 10 nm thick SiO*_x_* layer by thermal evaporation under vacuum conditions. A similar investigation has been reported in a recent publication about surface-enhanced fluorescence of fluorescent dyes on silica-protected Au nanoantennas by Fayyaz et al. [21], where the silica film thickness was varied between 5 and 25 nm.

**Figure 22:**
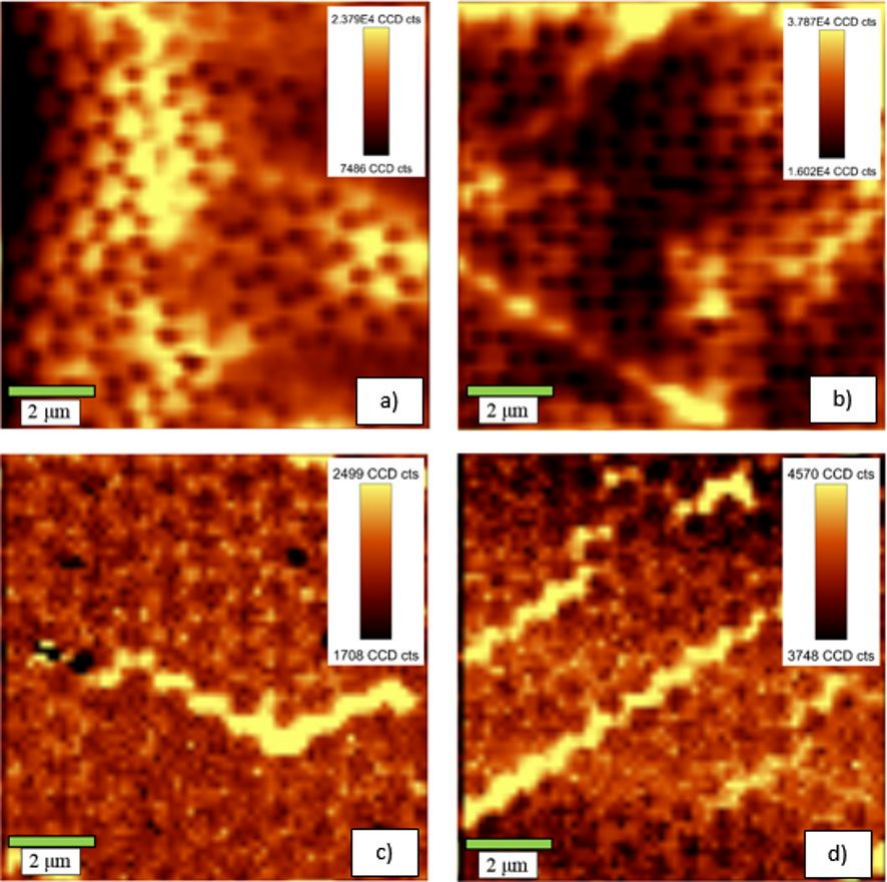
Representative fluorescence images (recorded at 614 nm) of samples without SiO*_x_* layer (a, b) and samples with a 10 nm SiO*_x_* layer deposited before spin coating with VTLUNP nanosuspensions (c, d).

The resulting fluorescence images of our samples for excitation with 532 nm radiation are shown in Figure 22 in comparison with the fluorescence images of noncoated samples. The fluorescence images of the samples coated with SiO*_x_* prior to spin coating of the VTLUNP solution (Figure 22c and Figure 22d) appear to be inverted when compared to the images without SiO*_x_* layer (Figure 22a and Figure 22b): one can see the pattern of the nanoantennas as bright spots in Figure 22c and Figure 22d, whereas the circular regions in between appear darker. The results suggest that the quenching may be the dominant effect when no dielectric spacer layer is applied. Further investigations with varying thickness of the SiO*_x_* layer will be undertaken to clarify this phenomenon in detail. We may also expect similar quenching effects in the spin-coated TiO_2_:Eu layers. However, since strong quenching is only expected for fluorescent particles in close contact with the metallic surfaces, the quenching effects may not be so obvious in the TiO_2_:Eu due to a large roughness of these layers as compared to the smoother VTLUNP layers (as deduced from a direct comparison of the AFM images in Figure 16 and Figure 20).

## Conclusion

Systematic studies of energy transfer in hybrid metal-oxide nanostructures have been performed. The main aim was to study local electrical-field enhancements at plasmonic Ag nanoantennas by using nanophosphors with a large shift between excitation and emission wavelength. For this purpose, we have successfully synthesized TiO_2_:Eu nanophosphors, which may be used as local field probes on the nanoscale. The optical properties of the nanophosphors indicate that ion-to-ion energy transfer plays a significant role in these systems. In addition, we have demonstrated that potentially passivating coatings with stable oxide layers can be achieved by postprocessing the nanophosphors with ALD. The TiO_2_:Eu nanophosphors have been tested in combination with well-defined periodic arrays of triangular Ag nano-antennas, which have been produced by nanosphere lithography. The optical properties of the Ag nano-antennas have been investigated by numerical simulations based on solutions of Maxwell’s equations. We find large resonant enhancements of the electrical field in the vicinity of the antennas, especially in the gaps of the bowtie structures. The magnitude of the field enhancement depends sensitively on the local environment of the antennas and the polarization of the incident light. In particular, we find that the presence of the nanophosphor layer itself has a significant effect on the resonances. We conclude that a well-defined arrangement of the nanophosphors is a necessary prerequisite for a quantitative determination of the energy transfer from fluorescence measurements. Based on the simulation results, we investigated procedures for the optimization of the nanophosphor layer structure. The approach used here is spin coating of nanosuspensions containing phosphor particles. Initial attempts with spin-coated TiO_2_:Eu nanophosphors show that large aggregates do still form in some places, although the general particle arrangement can be improved. It is demonstrated that these aggregates lead to high local fluorescence yields, which make a quantitative interpretation of the measured fluorescence intensities difficult. Better homogeneity of the fluorescent particle layer has been obtained by spin coating the nanoantennas with a commercial Eu-based organic fluorescent nanosuspension with narrow size distribution and similar optical properties. Quenching of the Eu fluorescence at the Ag nanoantennas has been observed by confocal microscopy of the fluorescent emission. The quenching can be reduced by deposition of a 10 nm thick SiO*_x_* spacer layer. In the systems containing this spacer layer, local enhancements of the fluorescence intensity at the nanoantenna positions can be observed.

In summary, the results show the potential for nanophosphors as local probes for energy transfer in hybrid metal-oxide nanosystems. A quantitative measurement of the energy transfer processes requires precise control of the geometry of both metal and oxide constituents of the system, and the suppression of quenching effects. In addition, numerical modeling of the electromagnetic field distribution is most important due to the dependence of the resonant plasmonic behavior on the local environment of the nanoantennas.
